# Natural Killer KIR3DS1 Is Closely Associated with HCV Viral Clearance and Sustained Virological Response in HIV/HCV Patients

**DOI:** 10.1371/journal.pone.0061992

**Published:** 2013-04-16

**Authors:** Antonio Rivero-Juarez, Rafael Gonzalez, Angela Camacho, Barbara Manzanares-Martin, Antonio Caruz, Antonio Martinez-Peinado, Julian Torre-Cisneros, Juan A. Pineda, José Peña, Antonio Rivero

**Affiliations:** 1 Infectious Diseases Unit, Instituto Maimonides de Investigacion Biomedica de Cordoba (IMIBIC), Hospital Universitario Reina Sofia, Cordoba, Spain; 2 Immunology Service, Instituto Maimonides de Investigacion Biomedica de Cordoba (IMIBIC), Hospital Universitario Reina Sofia, Cordoba, Spain; 3 Immunogenetics Unit, Faculty of Sciences, Universidad de Jaen, Jaen, Spain; 4 Molecular Genetics Laboratory, Instituto Maimonides de Investigacion Biomedica de Cordoba (IMIBIC), Hospital Universitario Reina Sofia, Cordoba, Spain; 5 Infectious Diseases and Microbiology Unit, Hospital Universitario de Valme, Seville, Spain; Simon Fraser University, Canada

## Abstract

**Aim:**

To evaluate the influence of the presence of the killer cell immunoglobulin-like receptor (KIR) 3DS1 on HCV treatment response in HIV/HCV genotype 1 co-infected patients

**Methods:**

HIV/HCV co-infected patients were included. KIR3DS1, their specific HLA-B ligands and IL28B gene were genotyped. Reductions of plasma HCV RNA levels between baseline and week 1, week 2 and week 4 were analyzed for IL28B genotype and KIR3DS1 (HLA Bw4 or Bw6). Rapid and sustained virological response (RVR and SVR) rates were also analyzed.

**Results:**

Sixty HIV/HCV genotype 1 co-infected patients were included. Patients with KIR3DS1 and Bw4 had higher rates of HCV viral decline than those who were not carriers of KIR3DS1 (week1: p = 0.01; week2: p = 0.038; week 4: p = 0.03). Patients carrying KIR3DS1/Bw4 had higher rates of RVR and SVR than those who did not carry KIR3DS1 (RVR: 46.15% *versus* 17.02%, p = 0.012; SVR: 63.6% *versus* 13 26.5%, p = 0.031). With respect to patients carrying the IL28B-CC genotype, those with KIR3DS1/Bw4 had greater rates of HCV viral clearance (week1: p<0.001; week2: p = 0.01; week 4: p = 0.02), RVR (p = 0.015) and SVR (p = 0.029) than those not carrying KIR3DS1.

**Conclusion:**

Our results show that the KIR3DS1 genotype has a positive effect on HCV viral clearance during the first weeks of Peg-IFN/RBV treatment in HCV/HCV co-infected patients bearing genotype 1, and higher RVR and SVR rates.

## Introduction

In recent years, the IL28B rs12979860 polymorphism has been identified as the best baseline predictor of sustained virological response (SVR) in both HCV monoinfected and HIV co-infected patients bearing genotype 1 [1], [2]. The mechanism of action of the IL28B gene remains unknown. We do know however that its beneficial impact on HCV viral clearance is due to a greater and more rapid HCV viral decline in the first weeks following start of treatment with pegylated-interferon (Peg-IFN) plus ribavirin (Peg-IFN/RBV) [3], [4]. It has been hypothesized that this beneficial impact is due to the fact that patients with the IL28B-CC genotype are more susceptible to exogenous IFN administration than those with the IL28B non-CC genotype [5]. However, the association between interferon-stimulated gene (ISG) expression and the IL28B genotype is a controversial point [6]. This suggests that there may be other factors that modify the effect of the IL28B genotype on HCV treatment response [7], [8].

The IL28B-CC genotype has been associated with higher rates of spontaneous resolution of acute HCV infections, and so with lower proportions of chronically infected patients [9]. This point suggests that the IL28B mechanism of action could be related, at least in part, to some immunological component. More specifically, it has been suggested that there could be a close relation between IL28B and the expression of certain receptors present in Natural Killer (NK) cells [6].

NK cells are the most prevalent lymphocyte in the liver and they play an important role in innate immune response [10]. It has been reported that a decrease of intrahepatic and peripheral blood NK cells in the progression of HCV infection [11] leads to deficiencies in activation which would favor disease chronicity. HCV treatment, however, seems to lead to a massive activation of the innate immunity response [12], [13] due to the fact that IFN-α is a potent activator of NK cells [14]. Natural killer cell immunoglobulin-like receptors (KIRs) are receptors on the NK cell surface associated either with activating (with short [*S*] cytoplasmic tails) or inhibiting (with long [*L*] cytoplasmic tails) NK cell action [15]. The presence of several KIRs has been associated with the outcome of HCV virus treatment (2DL2, 2DL3, 2DS1 2DS2 and 3DL1) [16], [17]. The expression of *S*-KIR on the NK cell surface promotes cytolysis and IFN-gamma production against target cells expressing the specific major histocompatibility complex class I ligand [15], [18], [19]. So, NK cell function is tuned by the interaction of NK cell receptors, such as KIR, with their ligands, so that, in order to evaluate the possible influence of KIRs on treatment response, it is necessary to determine the presence of its specific ligand.

KIR3DS1, responsible for activating NK cells, has been described as having a protective role against the development of hepatocellular carcinoma in HCV infected patients [19]. However, its impact on HCV treatment outcome has not been described.

The aim of our study therefore was to evaluate the influence of KIR3DS1 variants on HCV viral decline in HIV/HCV genotype 1 co-infected patients in the first weeks after the start of Peg-IFN/RBV treatment.

## Methods

### Ethical aspects

The study was designed and performed according to the Helsinki Declaration and approved by the ethical committee of the Reina Sofía University Hospital, Cordoba, Spain. All patients provided written informed consent before participating in this study.

### Patients

Caucasian HIV-infected patients with chronic hepatitis C bearing genotype 1, naïve to HCV treatment and receiving a Peg-IFN/RBV combination therapy, were included in this prospective study. The criteria used to determine hepatitis C therapy followed international guidelines [20]. Host, clinical and virologic characteristics were collected. Fibrosis stage was determined by biopsy or liver transient elastography (FibroScan®, Echosen, Paris). Significant fibrosis was defined as a METAVIR fibrosis score of F3-F4 in liver biopsy or a liver stiffness value of ≥11 kPa.

### Treatment regimens

All individuals were treated with Peg-IFN α2a, at doses of 180 µg, combined with a weight-adjusted dose of oral ribavirin (1000 mg/day for <75 kg, 1200 mg/day for ≥75 kg), in accordance with international guidelines [20], and completed at least 4 weeks of treatment.

### Virologic evaluation and definition of treatment response

Plasma HCV RNA loads were measured at baseline and at weeks 1, 2 and 4, using a quantitative PCR assay (Cobas TaqMan, Roche Diagnostic Systems Inc., Pleasanton, CA, USA), with a detection limit of 15 IU/mL.

SVR was defined as an undetectable serum HCV RNA level at 24 weeks after completion of HCV therapy. Undetectable plasma HCV RNA at week 4 was considered a rapid virological response (RVR).

### Single Nucleotide Polymorphism (SNP) genotyping

DNA was extracted using the automated MagNA Pure DNA extraction method (Roche Diagnostics Corporation. Indianapolis, IN 46250, USA). SNP rs129679860, located 3 kilobases upstream of the IL28B, and in strong linkage disequilibrium with a non-synonymous coding variant in the IL28B gene (213A>G, K70R; rs81031142), was genotyped. Genotyping was carried out using a custom TAQMAN assay (Applied Biosystems, Foster City, California, USA) on DNA isolated from whole blood samples, using a Stratagene MX3005 thermocycler with MXpro software (Stratagene, La Jolla, California, USA), according to manufacturer' instructions. The researchers responsible for genotyping were blinded to other patient data. The IL28B genotype was defined as CC or non-CC (TT/CT).

### KIR genotyping

KIR genotyping was performed using sequence-specific primers able to detect the presence of 16 different KIR genes previously used by Gomez-Lozano *et al* [21]. This method provided a high degree of resolution, since each primer pair identifies two linked, *cis-*located polymorphic sites (Invitrogen Corporation).

### Human leukocyte antigen (HLA)–B genotyping

HLA-B genotyping was performed with the INNO-LIPA HLA-B Multiplex kit (Innogenetics N.V.), using HLA-B multiplex primers for nucleic acid amplification of the second to the fourth exon of the HLA-B locus, and HLA-Bw4 primers for exon 2 of the HLA-Bw4 alleles. This is based on the PCR-SSO reverse method. Next, HLA-B alleles and Bw4 or Bw6 specificities were determined with LIRASTM software for INNO-LIPA HLA. Patients carrying KIR3DS1 were defined on the basis of specific ligands Bw4/Bw4 or Bw4/Bw6 (KIR3DS1/Bw4) or Bw6/Bw6 (KIR3DS1/Bw6) [22].

### Statistical Analysis

Continuous variables were expressed as mean±standard deviation or median and quartiles (Q1-Q3) and were analyzed using the Student's *t* test, the Mann-Whitney *U*-test or the Kruskal-Wallis test. Categorical variables were expressed as number of cases (percentage). Frequencies were compared using the χ^2^ test or Fisher's exact test. Significance was set at a *p* value of less than 0.05. Reductions in plasma HCV RNA were analyzed for IL28B and KIR3DS1 between baseline and weeks 1, 2 and 4. RVR and SVR rates were analyzed in terms of KIR3DS1 and IL28B genotypes. For the purpose of this analysis, SVR was assessed in an on-treatment approach, excluding those who voluntarily dropped out or discontinued therapy due to an adverse event. A linear regression model of HCV viral decline between baseline and weeks 1, 2 and 4 was performed. The analysis was performed using the SPSS statistical software package, version 18.0 (IBM Corporation, Somers, NY, USA).

## Results

### Baseline patient characteristics

Sixty HIV/HCV genotype 1 co-infected patients were included in this prospective study. Baseline characteristics are shown in Table 1. 21 (35%) patients carried the IL28B-CC genotype and 39 (65%), the IL28B non-CC genotype.

**Table 1:** Baseline population characteristics.

Characteristics	
N	60
Age (years) mean±SD	43±4.7
Sex (male), n (%)	53 (88.3)
Undetectable HIV viral load, n (%)	58 (96.6)
Use of HAART, n (%)	58 (96.6)
CD4 cell count (cells/mm^3^), mean±SD	487±240
AIDS criteria in the past, n (%)	13 (21.6)
HCV baseline viral load (log_10_ IU/mL), mean±SD	6.06±0.93
Significant liver fibrosis stage, n (%)	33 (55)
Liver cirrhosis, n (%)	21 (35)
Platelet count (10^3^/ µL), mean±SD	172.65±71.08
Total cholesterol levels (mg/dL), mean±SD	167±40
LDL cholesterol (mg/dL), mean±SD	87±33
HDL cholesterol (mg/dL), mean±SD	41±13.5
Triglycerides (mg/dL), mean±SD	179±93

Legends: standard deviation (SD), highly active antiretroviral treatment (HAART), human immunodeficiency virus (HIV), acquired immunodeficiency syndrome (AIDS), hepatitis C virus (HCV), low-density lipoprotein (LDL), high-density lipoprotein (HDL).

Twenty-four (40%) patients had KIR3DS1, and 36 (60%) did not. Among patients with KIR3DS1, 9 (37.5%) had Bw4/Bw4, 4 (16.6%) Bw4/Bw6 and 11 (45.9%) Bw6/Bw6. The distribution of KIR3DS1 and HLA-B by IL28B genotype is shown in Table 2. In our study, 38.6% of patients carrying the IL28B-CC genotype had KIR3DS1/Bw4, whereas only 17.9% of non-CC patients had KIR3DS1/Bw4 (p = 0.016).

**Table 2:** Distribution of patients by IL28B genotype, according to the presence or absence of KIR3DS1 and its ligands (Bw4 or Bw6).

	KIR3DS1			
	Absent	Bw4/Bw4	Bw4/Bw6	Bw6/Bw6
IL28B-CC. N (%)	15 (71.4)	5 (23.8)	1 (4.8)	0
IL28B non-CC. N (%)	21 (53.8)	3 (7.6)	4 (10.2)	11 (28.4)

Legends: interleukin 28B (IL28B), number of patients (N).

### Influence of KIR3DS1 and IL28B on HCV viral decline

**Figure 1:**
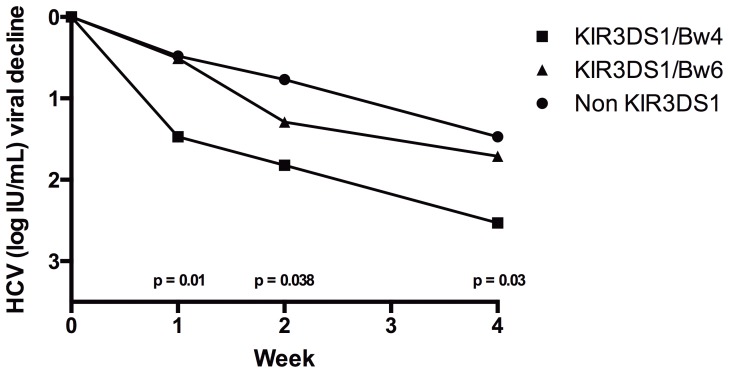
HCV viral decline for KIR3DS1 and its ligands during the first weeks of treatment with Peg-IFN/RBV.

As shown in Figure 1, patients with KIR3DS1/Bw4 showed greater HCV viral decline than those who did not carry KIR3DS1 or who had KIR3DS/Bw6, at week 1 (KIR3DS1/Bw4: 1.47±0.49; KIR3DS1/Bw6: 0.508±0.41; No KIR3DS1: 0.48±0.37 UI log_10_/mL, p = 0.01), week 2 (KIR3DS1/Bw4: 1.82±0.47; KIR3DS1/Bw6: 1.29±0.61; No KIR3DS1: 0.77±0.49 UI log_10_/mL, p = 0.038) and week 4 (KIR3DS1/Bw4: 2.53±0.56; KIR3DS1/Bw6: 1.71±0.74; No KIR3DS1: 1.47±0.81 UI log_10_/mL, p = 0.03). A key point of our results is that there were no differences, at any time point analyzed, between patients who carried KIR3DS1/Bw6 and those who did not have KIR3DS1 (week1: p = 0.527; week2: p = 0.173; week 4: p = 0.358) (Figure 1).

Carriers of the IL28-CC genotype showed greater reductions in plasma HCV RNA levels than non-CC genotype carriers at every time point analyzed (week 1: 0.98±0.72 *versus* 0.39±0.51 UI log_10_/mL, p = 0.017; week 2: 1.67±0.81 *versus* 0.83±0.77 UI log_10_/mL, p = 0.028; week 4: 2.28±0.62 *versus* 1.47±1.06 UI log_10_/mL, p = 0.037).

### Influence of both IL28B genotype and KIR3DS1/Bw4 on HCV viral clearance

**Figure 2:**
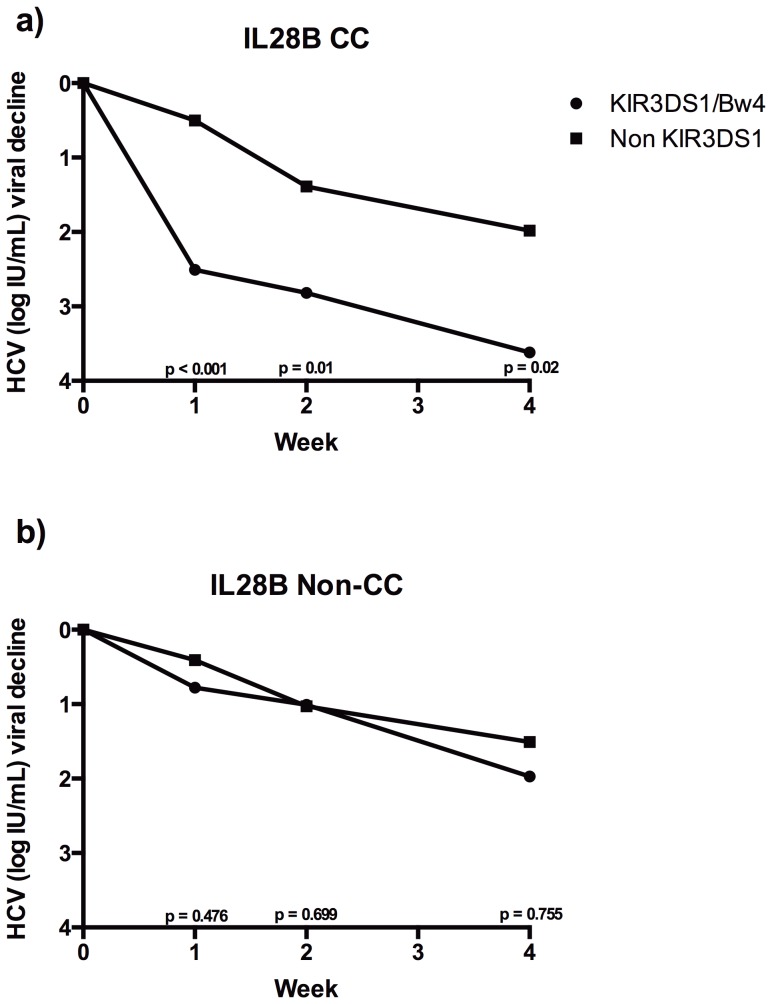
HCV viral decline for KIR3DS1 and its ligands in patients carrying the IL28B-CC (2A) or IL28B non-CC genotypes (2B).

Among carriers of the IL28B-CC genotype, those with KIR3DS1/Bw4 had higher rates of HCV viral clearance than those who were not KIR3DS1/Bw4 carriers (week 1: 2.51±0.67 *versus* 0.501±0.41 UI log_10_/mL, p<0.001; week 2: 2.82±0.73 *versus* 1.39±0.72 UI log_10_/mL, p = 0.01; week 4: 3.62±0.78 *versus* 1.98±0.98 UI log_10_/mL, p = 0.02) (Figure 2A). However, among IL28B non-CC genotype patients, no differences of HCV viral clearance were found irrespective of whether they carried KIR3DS1/Bw4 or not (week 1: 0.78±0.57 *versus* 0.41±0.39 UI log_10_/mL, p = 0.476; week 2: 1.01±0.67 *versus* 1.03±0.82 UI log_10_/mL, p = 0.699; week 4: 1.97±0.86 *versus* 1.51±0.84 UI log_10_/mL, p = 0.755) (Figure 2B).

When HCV viral clearance of patients carrying KIR3DS1/Bw4 was compared, those with the IL28B-CC genotype had higher rates of viral decline than those with the non-CC genotype (week 1: p<0.001; week 2: p = 0.03; week 4: p = 0.037). On the other hand, when we compared patients who did not carry KIR3DS1/Bw4, we did not find any differences of HCV viral clearance in the first weeks of treatment on the basis of IL28B genotype (week 1: p = 0.827; week 2: p = 0.725; week 4: p = 0.429).

The linear regression models showed that having KIR3DS1/Bw4, the IL28B-CC genotype and a low baseline HCV viral load were independent predictors of HCV viral decline between baseline and weeks 1, 2 and 4 (Table 3).

**Table 3:** Linear regression models for HCV viral decline from baseline to weeks 1, 2 and 4.

Variable	Condition	B	p
Baseline-Week 1			
IL28B	*CC*	0.617	0.026
KIR3DS1	*Bw4*	0.684	0.005
Baseline HCV viral load		−0.287	0.038
Liver Fibrosis	*F3*–*F4*	−0.141	0.589
Baseline-Week 2			
IL28B	*CC*	0.842	0.001
KIR3DS1	*Bw4*	0.813	0.002
Baseline HCV viral load		−0.433	0.004
Liver Fibrosis	*F3*–*F4*	−0.594	0.038
Baseline-Week 4			
IL28B	*CC*	1.1	0.002
KIR3DS1	*Bw4*	0.912	0.007
Baseline HCV viral load		−0.497	0.016
Liver Fibrosis	*F3*–*F4*	−0.456	0.222

Legend: adjusted coefficient (B), interleukin 28B (IL28B), hepatitis C virus (HCV).

### Treatment response rate

Eleven (18.3%) patients discontinued treatment due to abandoning therapy or adverse events. Of these patients, 8 carried IL28B non-CC (5 did not carry KIR3DS1, 2 had KIR3DS1/Bw6 and 1 had KIR3DS1/Bw4) and 3 carried IL28B-CC (2 did not carry KIR3DS1 and 1 had KIR3DS1/Bw4). Patients carrying KIR3DS/Bw4 achieved higher RVR and SVR rates than those who did not carry KIR3DS1/Bw4 (RVR: 6 [46.15%] *versus* 8 [17.02%], p = 0.012; SVR: 7 [63.6%] *versus* 13 [26.5%], p = 0.031). Patients with the IL28B-CC genotype had higher rates of RVR and SVR than those with the non-CC genotype (RVR: 8 [38.01%] *versus* 5 [12.8%], p = 0.03; SVR: 10 [55.5%] *versus* 10 [32.2%], p = 0.027). Patients carrying both the IL28B-CC genotype and KIR3DS1/Bw4 alleles had higher RVR and SVR rates than patients carrying only one or neither of these (Table 4).

**Table 4:** Treatment response by IL28B genotype and KIR3DS1/Bw4.

IL28B	3DS1/Bw4	RVR	P	SVR	P
*Non-CC*	*Yes*	1 (14.2%)	0.781	2 (33.3%)	1
	*No*	4 (12.5%)		8 (33.3%)	
*CC*	*Yes*	5 (83.3%)	0.015	5 (100%)	0.029
	*No*	3 (20%)		5 (38.4%)	

Legend: interleukin 28B (IL28B), rapid virological response (RVR), sustained virological response (SVR).

## Discussion

Our results show that the KIR3DS1 genotype had a positive effect on HCV viral clearance during the first weeks of Peg-IFN/RBV treatment in HCV/HCV co-infected patients bearing genotype 1, leading to higher rates of RVR and SVR. Our results also suggest that the beneficial impact of IL28B-CC on HCV treatment response may be enhanced by the presence of KIR3DS1.

NK cells play an important role in the outcome of several inflammatory diseases and contribute to the spontaneous resolution of infection [10]. The mechanism involves the activation of NK cells via endogenous IFN-γ production. Since HCV viral treatment is based on administering IFN-α, a dose of IFN-α activates NK cell cytotoxicity and improves HCV clearance [12], [13]. The presence of a specific KIR on the surface of the NK cell or its specific ligand on the hepatocyte surface could therefore increase or reduce the elimination of infected HCV cells and, in consequence, modify HCV viral decline. In this respect, the presence of some inhibitor KIRs (2DL1, 2DL3 and 3DL1) on the NK cell surface has been associated with weak HCV viral decline [16], [17], [23]–[25]. The ligand for KIR3DS1 has not been clearly identified, since no experimental evidence has been reported [19], [22]; even though Martin *et al* found an association between HLA-Bw4 and KIR3DS1 [26], there is still no consensus about the matter. Nonetheless, since KIR3DS1 and KIR3DL1 share a high degree of amino acid similarity in their extracellular domains, they might be expected to share a similar set of ligands [27]. In our study, we tested KIR3DS1, which participates in activating NK cells, and found that those patients who carried it showed greater HCV viral decline, leading to higher viral response rates, than those who did not.

In our study, the positive effect of the IL28B-CC genotype on HCV viral clearance during the first weeks of treatment was closely associated with KIR3DS1/Bw4. In this respect, patients carrying the IL28B-CC genotype had high HCV viral clearances only in the presence of KIR3DS1. In contrast, we found that, among those carrying the IL28B non-CC genotype, it made no difference whether they carried KIR3DS1 or not, in terms of influence on viral clearance. This is an important point because it tends to suggest that the positive effect of the IL28B-CC genotype on HCV treatment response could be conditioned by innate immunological status. This finding coincides with a recent report by Naggie *et al*, who reported that patients carrying the IL28B-CC genotype have a superior innate immune response to IFN-therapy than IL28B non-CC genotype patients [6]. These findings would be consistent with the accepted IL28B mechanism (endogenous-IFN activity), in the sense that, in chronic HCV infection, NK cells exhibit a polarized NK cell phenotype with decreased IFN-γ production [12]. This also supports the finding that patients carrying the IL28B-CC genotype have lower endogenous IFN activity than those with the non-CC genotype. Thus, a higher, but inadequate, endogenous IFN activity would lead to suboptimal stimulation of NK cells and a refractory effect when exogenous IFN was added [12]. This activity might condition the response to IFN-based therapy, depending on the host's baseline characteristics. So, determining the IL28B genotype alone would have limited power, since it could be conditioned by NK activation, regulated, at least in part, by KIR3DS1.

KIR3DS1, on the other hand, has been associated with slowing disease progression in HIV infection [28]–[31]. For this reason, we cannot rule out the possibility that patients with KIR3DS1 may have both an innate and adaptive immune system that is less negatively impacted by HIV infection, and so have a stronger immune response to HCV treatment than patients who are not carriers of KIR3DS1. However, the observed effect of KIR3DS1 on HCV treatment response could be due to the relative absence of KIR3DL1, rather than the presence of KIR3DS1. KIR3DL1 play an inhibitory role on NK cell activity. The functional union of KIR3DL1 with HLA-Bw4 triggers a “*do not eat*” reaction in NK cells with respect to the target cell [32]. One study showed that highly expressed KIR3DL1 alleles were beneficial in HIV disease and suggested that indirect effects on HIV could be the cause [33]. Consequently, if this effect is independent of HIV co-infection, the absence of KIR3DL1 alleles may release inhibition, resulting in more efficient lysis of infected hepatocytes in a KIR3DS1-independent manner.

The standard of care HCV treatment is due to change for HIV/HCV genotype 1 patients in the coming years [34]. The incorporation of the new protease inhibitors (PIs) to Peg-IFN/RBV will improve the rate of treatment response in this group of patients, as they did with HCV monoinfected patients [35], [36]. However, in the particular case of HIV protease inhibitors, interactions with antiretroviral treatment will have to be added to the drawbacks of the new PIs, along with a higher rate of adverse events and the cost of HIV/HCV co-infected patients [37]. This will be a key aspect of HCV treatment for HIV co-infected patients, since some will need to switch antiretroviral treatment, which has various clinical limitations. This is why identifying HIV/HCV co-infected patients who will respond to Peg-IFN/RBV therapy is a key feature of HIV clinical practice. Our results show that the major impact of NK cells, principally KIR3DS1, is on HCV viral decline during the first week of treatment. These findings could be used to optimize the choice of the most appropriate therapy for HIV/HCV co-infected patients bearing genotype 1 and to enhance the value of IL28B determination. Our findings could enable clinicians to detect patients with higher or lower probabilities of responding to Peg-IFN/RBV therapy.

However, our study has several limitations. Firstly, this study is a preliminary investigation since it included a small number of patients and did not have the statistical power to detect differences of KIR3DS1 among patients carrying the IL28B non-CC genotype. A larger cohort of HIV/HCV genotype 1 co-infected patients is required to analyze this. Secondly, our study determined only KIR3DS1 and rs12979860 when considering the respective impacts of NK cell KIR and IL28B. Studies analyzing the synergistic effect of other known KIRs (3DL3, 2DS2, 2DL2, 2DL3, 2DL5, 2DS3, 2DS1, 3DL2, 2DP1, 2DL1, 3DP1, 2DL4, 3DL1, 2DS5 and 2DS4) and IL28B (rs8099917) are needed. Thirdly, Bw4 alleles (Bw4*80I and *80T) were not considered in our study, which may be a limitation since these variations could play a role in conditioning the affinity of KIR3DS1 for Bw4. A fourth limitation is that this study included only HIV/HCV co-infected patients, which represents a unique population. In fact, HIV/HCV co-infected subjects attain SVR less frequently than HCV monoinfected individuals and their HCV viral decline is slower [38]. Hence, further studies of the predictive yield of KIR3DS1 variants are needed, which include HCV monoinfected patients. Finally, in our study, every patient carrying the IL28B-CC genotype with KIR3DS1 had HLA-Bw4, whereas no patient carrying the IL28B-CC genotype with KIR3DS1 had HLA-Bw6. It is not known whether there is a possible association between these factors. It is likely, however, that our observation was due to the small number of patients. A larger cohort would clarify these findings.

In conclusion, our study shows that KIR3DS1 has a positive impact on HCV viral clearance in Peg-IFN/RBV treatment in HIV/HCV co-infected patients bearing genotype 1, and this may be associated with the IL28B mechanism of action in HCV treatment response.
